# Evidence-based orthopedics and the myth of restoring the anatomy

**DOI:** 10.1080/17453674.2021.1916686

**Published:** 2021-04-23

**Authors:** Stig Brorson

**Affiliations:** Centre for Evidence-Based Orthopaedics, Department of Orthopaedic Surgery, Zealand University Hospital, Denmark, and Department of Clinical Medicine, University of Copenhagen, Denmark

As orthopedic surgeons we have a strong inclination towards bringing broken bones together. Traditionally, in displaced fractures the anatomy should be restored and the success of surgery should subsequently be documented by postoperative imaging. In some common upper limb fractures, for example in displaced fractures of the proximal humerus, best evidence challenges our intuitions. On the one hand, current evidence has failed to demonstrate any benefits to patients in bringing the displaced fragments together by means of plating or nailing or even by replacing the joint (Aspenberg 2015). The only difference is an increased risk of additional surgery in the surgical group (Handoll and Brorson 2015). On the other hand, by following evidence-based recommendations we shall face a substantial number of displaced fractures healing in malunion. Passed-down knowledge and practice are challenged.

As doctors we aim to offer the patient optimal treatment. Should this patient be offered surgery to restore the anatomy (Figure), even when randomized trials (Rangan et al. 2015, Launonen et al. 2019) have been unable to document any benefits to the patient in terms of function, quality of life, pain, or any other outcome?

**Figure:**
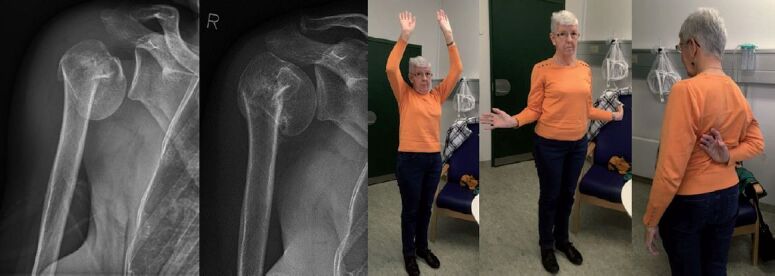
68-year-old female suffering an impacted 2-part fracture of the proximal humerus. Radiographs were taken on admission and after 5 months. Clinical photos were taken after 5 months. Pain-free shoulder function was obtained after 3 months. In this case intuition will tell most orthopedic surgeons to restore the anatomy, most likely by open reduction and internal fixation with a locking plate. Best evidence from randomized trials does not support this decision (Rangan et al. 2015, Launonen et al. 2019). How to act?

## David Sackett in orthopedics

Let’s revisit David Sackett’s (1934–2015) definition of evidence-based practice: Evidence-based practice includes the integration of best available evidence, clinical expertise, and patient values (Sackett 1997).

1st, best available evidence does not support surgery in this case. 2nd, clinical expertise is limited when it comes to severely displaced fractures managed nonoperatively. Experience is mainly surgical, and the vast majority of studies read in orthopedic departments are clinical series concerning surgical techniques and implants. 67% of the literature on proximal humerus fractures concerns operative treatments compared with 4% including nonoperative treatments (Slobogean et al. 2015). 3rd, patient values need to be explored. They are not accessible from radiographs or demographic data yet often form the basis for operative decisions. Many elderly patients with displaced fractures of the proximal humerus have limited interest in surgical interventions unless the surgeon states that this is the only way to regain function and quality of life. However, this answer is no longer compatible with best evidence. Surprisingly, and for unknown reasons, in many countries the use of surgery for fractures of the proximal humerus has increased for decades and seems to be continuing to increase (Huttunen et al. 2012, Sumrein et al. 2017, Sabesan et al. 2017, Jo et al. 2019, Klug et al. 2019).

## The challenge to the modern orthopedic surgeon

As orthopedic surgeons we need to reconsider current practice for certain common fracture patterns like 2-, 3-, and 4-part fractures of the proximal humerus. Similar considerations can be made for other upper limb fractures. In some displaced fractures of the clavicle (Lenza et al. 2019), the humeral shaft (Rämö et al. 2020), the olecranon (Chen et al. 2021), and the distal radius (Mulders et al. 2018) even severe radiological malunion seems to be well tolerated, at least in older adults.

Patient selection is crucial. We should ask for the patient’s values and preferences instead of focusing exclusively on radiographic appearance and surgical techniques as the basis for shared decision-making. Outcome measures should be patient reported as radiographic measures poorly reflect patient preferences.

Orthopedic surgeons conducting evidence-based practice should be aware that “bringing the bones together” may be intuitively right but in some cases is not supported by the best available evidence.

Before the era of radiology and evidence-based medicine the pioneer of surgery and surgical pathology, R.W. Smith (1807–1873), clearly stated the point:

*“The impacted fracture of the neck of the humerus always unites with a certain amount of deformity … it would be imprudent to restore to the joint its natural form, even were it in our power to accomplish it, for we would thus materially diminish the chance of the occurrence of osseous consolidation … but the prudent surgeon will never omit to announce to the patient that a certain degree of impairment of the motions of the joint will be a permanent result of the injury (*Smith 1847*).”*
